# TIPE3 is a candidate prognostic biomarker promoting tumor progression via elevating RAC1 in pancreatic cancer

**DOI:** 10.1186/s12943-022-01626-5

**Published:** 2022-08-09

**Authors:** Zequn Li, Shougen Cao, Yuqi Sun, Zhaojian Niu, Xiaodong Liu, Jun Niu, Yanbing Zhou

**Affiliations:** 1 Department of Gastrointestinal Surgery, The Affiliated Hospital of Qingdao University, Qingdao, 266000 Shandong China; 2 Department of General Surgery, Qilu Hospital of Shandong University, Jinan, 250012 Shandong China

## Main text

Pancreatic cancer (PC) is the most lethal solid tumor all around the world. Most of the PC patients always present unfavorable prognosis [1]. Although progress has been made in the comprehensive therapies of this devastating disease in recent decades, the absence of effective biomarkers still leads to poor prognosis [2, 3]. Surgical resection remains the main treatment, but nearly 70% ~ 80% patients with PC have been diagnosed as advanced or locally advanced stage. The possibility for radical resection for this tumor is merely 8% ~ 12%. Predicting prognosis based on resection alone is difficult [4, 5]. Therefore, exploring novel biomarkers for predicting the prognosis of PC patients has pivotal clinical value [6].

The TNFAIP8 (tumor necrosis factor-alpha-induced protein 8, or TIPE) family has been found to be a pivotal regulator of tumorigenesis [7]. Tumor necrosis factor-α-induced protein 8 like-protein 3 (TIPE3) is the latest described member of TIPE family, which presents high structural homology with the other members of TIPE family [7, 8]. TIPE3 was identified as a lipid transfer protein, which could interact directly with PtdIns (4, 5) P2 (PIP2). TIPE3 mainly exists in epithelial cells with secretory function. TIPE3 plays a pivotal role in apoptosis, cell proliferation and signal transduction [7, 9, 10]. As a second messenger transporter, TIPE3 is involved in the occurrence of several tumors. TIPE3 mainly served as tumor promotor and it has been reported to activate PI3K-AKT pathway as well as MAPK-ERK pathway [9, 11].

In this study, we sought to explore whether TIPE3 is a biomarker for PC and molecular targets for PC treatment.

## Materials and methods

### Retrospective cohort and database

The retrospective cohort composed of 188 PC patients that underwent surgical resections with R0 margin. The inclusion criteria: (1) formalin-fixed tumor tissues and adjacent normal tissues with detailed medical records, (2) no adjuvant chemotherapy or radiotherapy, (3) postoperative existing time longer than 1 month, (4) no history of other tumors. Following-up information until Dec 2014. Tumor staging was evaluated on the basis of the 8th edition of AJCC classification. The mRNA level of tipe3 was appraised via GEPIA database (http://gepia.cancer-pku.cn/).

### Prospective cohort

A prospective cohort was established from Jan 2015 to Dec 2017, which composed of 66 patients. The data was gained until Dec 2020. Written informed consent was obtained from each patient, and this study was approved by the Ethics Committee of Shandong University and the Ethics Committee of Qingdao University, China. (Approval number, KYLL-2015KS-114).

### Immunohistochemistry (IHC) and evaluation

IHC was performed and evaluated according to the procedure reported previously [12]. The tissue were immunostained with anti-TIPE3 polyclonal antibody (dilution 1:300, BOSTER, China) or anti-Rac1 monoclonal antibody (diluted 1:300, Abcam, UK). The cut-off point was verified using the X-tile program.

### Nomogram construction and validation

Univariate/multivariate Cox proportional hazards was constructed to estimate prognostic risk factors. A diagnostic prediction model was developed as a nomogram according to the independent prognostic factors of survival. To assess the model performance, the discrimination and calibration of the nomogram were performed. The discriminative power of the nomogram was computed by Harrell’s concordance index (C-index, range from 0.5–1.0). The calibration curve was used to measure the accuracy of the nomogram, while the 45-degree line was used as the optimal model. Furthermore, the Decision curve analysis (DCA) algorithm could be used as a comprehensive method to evaluate the clinical significance and net benefit of the predictive model [13].

### Cell culture

Human PC cell lines including AsPC-1, MIA PaCa-2, Capan-1, CFPAC-1, PANC-1, BxPC-3, Patu-8988 and SW-1990 were obtained from American Type Culture Collection and cultured in specified medium (DMEM, 1640, L-15 or IMDM) supplemented with 10% inactivated fetal bovine serum (FBS) (Gibco, CA, USA).

### Stable cell line construction

In order to stabilize TIPE3 expression, lentivirus mediated transfection was carried out in this study. PC cells were infected and stable shRNA-expressing or TIPE3-expressing cells were obtained by screening with ampicillin.

### qPCR

Total RNA was extracted by Trizol reagent (Invitrogen, Carlsbad, USA). Then, reverse transcription was performed using a ReverTra Ace qPCR Kit (Toyobo, Osaka, Japan). Ultra SYBR Mixture (CWBIO) was used in qPCR. Table S7 listed the primers used in the present study.

### Western blotting

PC cells were collected and lysed by RIPA cell lysis buffer and transferred to PVDF. Polyvinylidene fluoride membrane was hatched with primary antibodies against TIPE3 (dilution 1:300, BOSTER, China), RAC1 (dilution 1:300, Abcam, UK) or β-actin (1:1000, ZSGB-Bio, China) at 4 °C overnight. ECL Kit (Millipore, Bradford, USA) was estimated for development, and Image J software was used to evaluate band density.

### Cell proliferation assay

Cells were seeded at a density of 3000 cells per well. CCK8 solution was added and the absorbance value was measured at 450 nm after 2 hours.

### Transwell assay

Cell migration assays were analyzed by a 24-well Transwell system (Costar, Acton, USA). The cell suspension was maintained in the upper cavity of the Transwells. Cell invasion assays were conducted using Matrigel pretreated Transwell system as previously reported [12].

### Pancreatic cancer xenograft mouse model

The orthotopic xenograft tumor model was established using stably transfected PC cell lines. A total number of 5*10^6^ cells in a volume of 50 μl cell suspension was injected into the pancreas of 5–6 weeks male nude mice. A bioluminescent imaging was conducted to visualize the growth of orthotopically xenografted tumors dynamically. Solid tumors were separated and then processed for histopathological examination. The survival rate and distant metastasis were observed by repeating the aforementioned experiments with a prolonged duration of 90 days. All animal experiments were performed according to the National Institute of Health Guide for the Care and Use of Laboratory Animals and approved by the Scientific Investigation Board of Qingdao University (Qingdao, Shandong Province, China).

### Pancreatic cancer metastatic mouse model

The total number of 5*10^6^ stable transfected PC cells in 200 μl saline were injected into the spleen of mice. Bioluminescent imaging method was used for visualizing the dynamic metastatic conditions. All the mice were sacrificed after 7–9 weeks. Organs with metastatic tumors such as livers and lungs were separated. The number of macroscopic tumors were enumerated and metastatic tumors were proved via histopathological examination.

### Statistical analysis

The associations between TIPE3 expression and clinicopathological parameters were analyzed by the chi-square test or Fisher’s exact test. The cumulative OS rates were calculated by Kaplan–Meier method, and the statistical differences between subgroups were calculated by log-rank test. Independent prognostic factors were identified by multivariate analysis with Cox-regression model. The statistical comparisons between control and tested group were analyzed with the one-way, two-way ANOVA or *t* tests. All statistical analyses were performed using SPSS 18.0 software (SPSS Inc., Chicago, USA), and a *P* value < 0.05 was considered statistically significant.

## Results and discussion

### TIPE3 expression is up-regulated in PC specimens

**Fig. 1:**
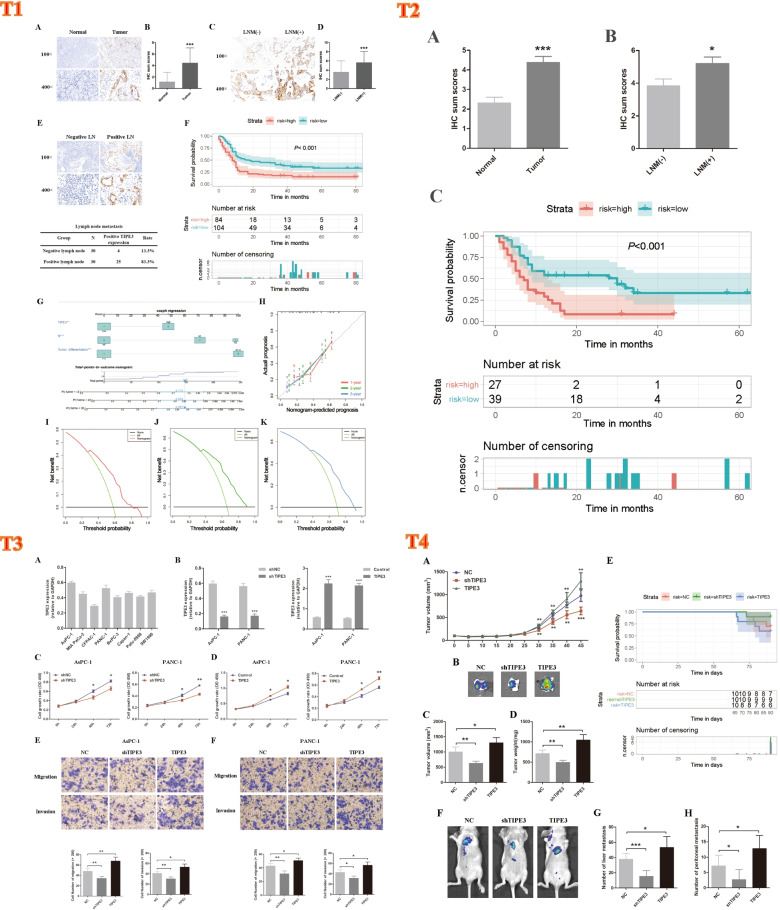
T1: The expression and clinical significance of TIPE3 in PC. **A** Representative IHC staining of TIPE3. **B** IHC sum scores were applied to assess TIPE3 in PC specimens. **C**) IHC staining of TIPE3 in PC tissues with or without lymph node metastasis. **D** IHC sum scores were applied to assess TIPE3 in PC specimens with or without lymph node metastasis. **E** TIPE3 expression in negative lymph node and metastatic lymph node. **F** Survival analysis according to TIPE3 expression in 188 PC patients. **G** Prognostic Nomogram of 188 PC patients. **H** Calibration curves of the OS nomogram. (I-K) DCA for the OS nomogram at 1-year **I** 2-year **J** and 3-year **K**. ***, *P* < 0.001. T2. The clinical significance of TIPE3 **A** IHC sum scores were applied to determine TIPE3 in PC. **B** IHC sum scores were applied to assess TIPE3 expression in PC specimens. **C** Survival analysis using TIPE3 in 66 PC patients. *, *P* < 0.05; ***, *P* < 0.001. T3. TIPE3 promoted malignant biological behaviors of PC cells. **A** Detection of TIPE3 mRNA expression using qRT-PCR. **B** TIPE3 expression were detected after TIPE3 silencing or overexpression using qRT-PCR. **C** CCK8 analysis was conducted after TIPE3 silencing in AsPC-1 and PANC-1 cells. **D** CCK8 analysis was conducted after TIPE3 overexpression in PC cells. **E** Trans-well assays were performed after TIPE3 silencing or overexpression in AsPC-1 cells. **F** Trans-well assays were performed after TIPE3 silencing or overexpression in PANC-1 cells. *, *P* < 0.05; **, *P* < 0.01; ***, *P* < 0.001. T4. TIPE3 promoted tumor progression and metastasis in mice. **A** Tumor growth curve of orthotopic xenograft mouse. **B** Representative pictures of primary tumors in pancreas. **B** Tumor volume in NC, shTIPE3 and TIPE3 group, respectively. **D** Tumor weight in NC, shTIPE3 and TIPE3 group, respectively. **E** Survival curves of different groups. **F** The representative of bioluminescent images in metastatic mouse model. **G** Number of liver metastatic foci were recorded. **H** Number of peritoneal metastatic tumors were recorded. *, *P* < 0.05; **, *P* < 0.01; ***, *P* < 0.001

TIPE3 mRNA expression were analyzed including 171 normal individuals and 179 patients with PC. Compared with normal tissues, TIPE3 mRNA was significantly raised in PC tissues. IHC results of 188 PC tissue specimens from the retrospective cohort also demonstrated that TIPE3 expression was increased in PC tissues. And immuno-staining found that TIPE3 mainly localized in both cytoplasm and membrane of PC cells (Fig. 1(T1) A-B).

The baseline characteristic of this cohort was shown in Table S1. The increased TIPE3 in tumor tissues was correlated with lymph node metastasis (*P* < 0.001) and TNM stage (*P* < 0.001) (Fig. 1 (T1) C-D, Table S1; Table S5). Moreover, we also detected TIPE3 expression in 30 negative lymph nodes and 30 metastatic lymph nodes, and results showed that positive TIPE3 rate was significantly higher in metastatic lymph nodes (Fig. 1 (T1) E).

### High TIPE3 expression is associated with poor survival of PC patients

To determine the role of TIPE3 in overall survival (OS) of PC patients, univariate analysis with K-M method was conducted. In this cohort, patients with advanced N stage (*P* < 0.001) and poor differentiation (*P* < 0.001) had lower OS. Advanced TNM stage (*P* = 0.067) also tend to indicate lower OS, although this tendency was not of statistical significance, which may results from limited number of stage III-IV patients (Table S2). Importantly, high TIPE3 expression (*P* < 0.001) also led to unfavorable prognosis.

Multivariate analysis was further performed and results found that high level of TIPE3 (*P* = 0.013) was an independent unfavorable prognostic factor (Table S2, Fig. 1(T1) F).

### Construction of the nomogram based on TIPE3 expression

According to the aforementioned results, tumor differentiation, 8th edition AJCC N stage, and TIPE3 expression was established to predict the overall survival rate (Fig. 1(T1) G). Results showed the C-index was up to 0.679 (95% CI: 0.630–0.728). The calibration curve for the OS probability at 1, 2, or 3-year showed favorable calibration of the nomogram (Fig. 1(T1) H). In addition, DCA of the nomogram indicated that the model had a favorable net clinical benefit for predicting survival rates (Fig. 1 (T1) I-K).

### Clinical significance of TIPE3 expression in the prospective cohort

Our previous results found that TIPE3 was closely correlated with the prognosis of PC using a retrospective cohort and nomogram analysis. We further investigated the expression and clinical significance of TIPE3 expression in PC using a prospective cohort that consisting of 66 PC patients. The expression of TIPE3 was also raised in tumor tissues, and elevated TIPE3 expression was associated with lymph node metastasis as well (*P* = 0.030) (Table S3, Fig. 1 (T2) A-B; Table S6). Then univariate and multivariate analysis were conducted, revealed that poor tumor differentiation (*P* = 0.002) and high TIPE3 level (*P* = 0.018) were closely associated with worse survival. Notably, high TIPE3 expression was also identified as an independent prognostic factor for PC patients in the prospective cohort (Table S4, Fig. 1(T2) C).

### TIPE3 accelerates tumor progression in vitro

Our previous results found that TIPE3 might be involved in the progression of PC. We observed that all the eight PC cells (AsPC-1, MIA PaCa-2, CFPAC-1, PANC-1, BxPC-3, Capan-1, Patu-8988 and SW-1990) presented moderate to high TIPE3 expression (Fig. 1 (T3) A). Specifically, we chose two cell lines (AsPC-1 and PANC-1) with highest TIPE3 expression to perform gene-knockdown experiments via lentivirus transfection of TIPE3-shRNAs. The knockdown and overexpression efficiency was testified (Fig. 1 (T3) B). The effect of TIPE3 on malignant behaviors of tumor cells was evaluated via CCK-8 assay and Transwell assay. Results showed that TIPE3 silencing attenuated the proliferation, migration and invasion capacities of PC cells. In addition, to testify the related conclusions, TIPE3 was overexpressed in PC cells, CCK-8 and Transwell assays were repeated. The proliferation, migration and invasion capacities were enhanced after TIPE3 overexpression (Fig. 1 (T3) C-F).

### TIPE3 promotes tumor progression in vivo

Orthotopic xenograft mouse model was established using stable TIPE3 silenced (LV-shTIPE3) or TIPE3 overexpressed (LV-TIPE3) AsPC-1 cells. Tumors from LV-shTIPE3 group showed slower growth, smaller size and lighter weight (Fig. 1 (T4) A-D). Mice from the shTIPE3 group also presented increased survival compared to controls (86.8 vs 88.3 days). While tumors from LV-TIPE3 group presented increased size and weight, and TIPE3 overexpression led to decreased survival rate (86.8 vs 83.4 days) (Fig. 1 (T4) E).

To evaluate the function of TIPE3 on the metastasis of PC, a metastatic mouse model was constructed. We found that mice in the LV-shTIPE3 group presented reduced number of distant metastasis compared with LV-shNC group. The size of metastatic tumors were also smaller in the LV-shTIPE3 group. While in the LV-TIPE3 group, the number and size of metastatic tumor were markedly increased compared to control (Fig. 1 (T4) F-H). All these results demonstrated that TIPE3 promoted tumor progression and metastasis in vivo.

### TIPE3 promoted RAC1 expression in PC

**Fig. 2:**
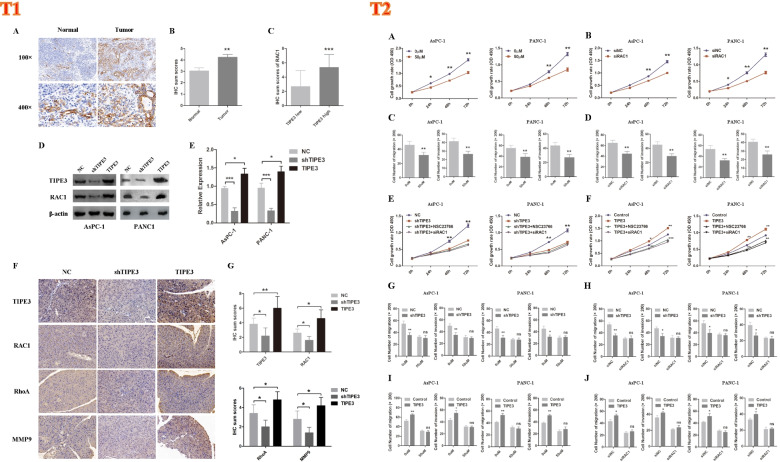
T1: TIPE3 increased RAC1 in PC. **A** IHC staining of RAC1. **B** IHC sum scores were applied to determine RAC1 expression. **C** IHC sum scores of RAC1 in PC tissues with low or high TIPE3 expression. **D** Western-blot was conducted to detected RAC1 expression after TIPE3 silencing or overexpression. **E** Quantified results of western-blot for RAC1 expression in PC cells. **F** IHC staining of TIPE3, RAC1, RhoA and MMP9 in orthotopic xenograft tumors. **G** IHC sum scores of TIPE3, RAC1, RhoA and MMP9 expression in orthotopic xenograft tumors. *, *P* < 0.05; **, *P* < 0.01; ***, *P* < 0.001. T2. TIPE3 accelerated the malignant behaviors of PC cells in a RAC1-dependent manner. **A** NSC23766 (50 μM) was used in CCK8 analysis. **B** CCK8 assays was measurement after RAC1 silencing in PC cells. **C** Trans-well assays after treatment of NSC23766 (50 μM). **D** Trans-well assays were measurement after RAC1 silencing in PC cells. **E** CCK8 assays were measurement in TIPE3 silencing PC cells that pretreated with NSC23766 (50 μM) or transfection with RAC1 siRNA. **F** CCK8 assays were conducted in TIPE3 overexpressed PC cells that pretreated with NSC23766 (50 μM) or transfection with RAC1 siRNA. **G** Trans-well assays were conducted in TIPE3 silenced PC cells that pretreated with NSC23766 (50 μM). **H** Trans-well assays were conducted in TIPE3 silenced PC cells that transfected with RAC1 siRNA. **I** Trans-well assays were conducted in TIPE3 overexpressed PC cells that pretreated with NSC23766 (50 μM). **J** Trans-well migration and invasion assays were conducted in TIPE3 overexpressed PC cells that transfected with RAC1 siRNA

RAC1 could participate in tumor progression, especially the metastasis of PC. Research also found that other members of TIPE family functions through interacting with RAC1. So we hypothesized that TIPE3 may promoted PC progression via targeting RAC1. Therefore, we conducted the following experiments. First, RAC1 expression was observed in PC specimens using IHC, and results showed that RAC1 expression was also raised in PC tissues compared to the normal tissues (Fig. 2 (T1) A-B). Importantly, RAC1 expression was closely correlated with TIPE3 expression in PC tissues (Fig. 2 (T1) C). Moreover, decreased RAC1 expression was shown in TIPE3 silencing PC cells, whereas elevated RAC1 expression was shown in TIPE3 overexpressed PC cells (Fig. 2 (T1) 4D-E). Furthermore, RAC1 expression was also closely associated with TIPE3 expression in xenograft tumor tissues. In addition, the expression of RhoA and MMP9, a RAC1 downstream target, were also detected, and results showed both the expression of RhoA and MMP9 expression were related with TIPE3 expression in tumor tissues (Fig. 2 (T1) F-G). Taken together, these results indicated that TIPE3 promoted RAC1 expression in PC.

### TIPE3 promotes tumor progression in a RAC1-dependent manner

To explore whether TIPE3 promoted tumor progression via activating RAC1, NSC23766 and RAC1 silencing were used in the subsequent experiments. Both treatments dramatically suppressed the malignant behaviors of PC cells (Fig. 2 (T2) A-D). Notably, TIPE3 knockdown decreased the proliferation, migration and invasion of PC cells, while NSC23766 treatment and RAC1 silencing eliminated this effect. Consistently, NSC23766 treatment and RAC1 silencing also blocked the impact of TIPE3 overexpression on the malignant behaviors in PC cells (Fig. 2 (T2) E-J). These data indicated that TIPE3 promoted PC progression in a RAC1-dependent manner.

Pancreatic cancer (PC) has a high degree of malignancy and poor prognosis. Identifying effective biomarker is essential for the precise stratification and the development of targeted therapies, which is crucial for improving the prognosis of these patients [14, 15]. For the first time, we demonstrated that TIPE3 served as an independent prognostic biomarker in PC, which would contribute to stratifying patients with high risk and poor prognosis. Mechanically, TIPE3 promoted the progression of PC via targeting RAC1. These finds identified a potential biomarker for prognostic prediction of PC, and preliminarily revealed the functions and related mechanism of TIPE3 in PC.

As the last discovered TIPE family members, TIPE3 was demonstrated to be a novel regulatory molecule in a number of tumors recently, but the function of TIPE3 in PC remains unknown, especially the correlation between TIPE3 expression and patients’ prognosis is unclear [16]. In the present study, we constructed both retrospective cohort and prospective cohort to explore the clinical value of TIPE3 in PC tissues. This study shows that the level of TIPE3 in PC tissue is increased, which was closely associated with lymph node metastasis and TNM stage. Metastatic lymph nodes showed significantly higher positive TIPE3 expression rate, indicating that tumor cells with positive TIPE3 expression had increased metastatic capacity. Of note, high TIPE3 expression served as an independent unfavorable prognostic factor for PC patients.

At present, most of the clinical decision that made for PC patients largely depend on the TNM system. Data from related randomized controlled trials or prospective studies are also limited [4, 5, 14]. This study established a nomogram for predicting the OS for PC patients based on TIPE3 expression. Moreover, to testify our conclusion, we constructed another prospective cohort with 66 patients. These results revealed that TIPE3 act as an ideal marker for predicting patients’ prognosis, which is an essential supplement to the oncogenic role of TIPE3 and provided novel theoretical basis for the progress of PC. Therefore, we strongly recommend a routine TIPE3 staining for PC tissues. We then further investigated the role of TIPE3 both in vivo and in vitro. It is worth noting that we constructed three in vivo models to prove that TIPE3 promotes tumor progression and metastasis in PC.

RAC1 is a key regulator during tumor progression. It has been reported that RAC1 hyperactivation and up-regulation is closely correlated with enhanced growth and metastasis in numerous tumors, including PC. RAC1 has become a standard for tumor stratification and a promising therapeutic target due to its crucial role in tumor progression [17, 18]. The present study also primarily demonstrated that TIPE3 promoted tumor progression via up-regulating RAC1 expression in PC.

Previous research demonstrated that another TIPE family member, TIPE2, was also involved in regulating the activity of RAC1. The N-terminal lysine and arginine residues are essential for the interaction between TIPE2 and RAC1 [19, 20]. All the four members of TIPE family were of high homology, but the expression and roles of different TIPE family members varies a lot. The N-terminus of TIPE family members might be crucial for their functions [8]. Therefore, clarifying the key domain that responsible for the functions of TIPE3 in PC is necessary in further study. In addition, a larger prospective cohort is needed to evaluate the sensitivity and specificity of TIPE3 expression in predicting the prognosis of PC patients.

## Conclusions

Taken together, using both retro-and prospectively collected PC patient cohorts, we present a potential biomarker for risk stratification and prognostic prediction for PC patients. Moreover, TIPE3 is crucial for promoting tumor progression and metastasis. For the first time, this study found that TIPE3 functions through up-regulating RAC1, suggesting that TIPE3 may serve as a promising target for the treatment of PC.

## Supplementary Information

**Additional file 1: Table S1.** Correlation between TIPE3 expression and clinical characteristics of PDAC patients (Retrospective cohort). **Table S2.** Univariate and multivariate Cox proportional hazard analyses of patients with PDAC (Retrospective cohort). **Table S3.** Correlation between TIPE3 expression and clinical characteristics of patients with PDAC (Prospective cohort). **Table S4.** Univariate and multivariate Cox proportional hazard analyses of patients with PDAC (Prospective cohort). **Table S5.** Baseline characteristics of patients with PDAC (Retrospective cohort). **Table S6.** Baseline characteristics of patients with PDAC (Prospective cohort). **Table S7.** The primers used for qPCR analysis.

## Data Availability

The data and materials supporting the conclusions of this study are included within the article and its additional files.

## Abbreviations

FBS: fetal bovine serum
PC: pancreatic cancer
IHC: immunohistochemistry
TIPE: tumor necrosis factor-alpha-induced protein 8
GEPIA: the Gene Expression Profiling Interactive Analysis
DAB: 3, 5-diaminobenzidine
DCA: the Decision curve analysis
CCK8: the Cell Counting Kit-8
