# Supplementary material for: TIPE3 is a candidate prognostic biomarker promoting tumor progression via elevating RAC1 in pancreatic cancer
Source: Mol Cancer. 2022 Aug 9;21:160. doi: 10.1186/s12943-022-01626-5 (PMC9361694; doi:10.1186/s12943-022-01626-5)
Supplement: Supplementary file 1 — Additional file 1: Table S1. Correlation between TIPE3 expression and clinical characteristics of PDAC patients (Retrospective cohort). Table S2. Univariate and multivariate Cox proportional hazard analyses of patients with PDAC (Retrospective cohort). Table S3. Correlation between TIPE3 expression and clinical characteristics of patients with PDAC (Prospective cohort). Table S4. Univariate and multivariate Cox proportional hazard analyses of patients with PDAC (Prospective cohort). Table S5. Baseline characteristics of patients with PDAC (Retrospective cohort). Table S6. Baseline characteristics of patients with PDAC (Prospective cohort). Table S7. The primers used for qPCR analysis. [file 12943_2022_1626_MOESM1_ESM.docx]

| **Table S1.** Correlation between TIPE3 expression and clinical characteristics of PDAC patients (Retrospective cohort) | | | | | | | |
| --- | --- | --- | --- | --- | --- | --- | --- |
| Characteristic | Number | TIPE3 expression | | χ2 value | *P*-value | TIPE3 Staining Score | *P*-value |
|  |  | High (%) | Low (%) |  |  |  |  |
| Age (years) |  |  |  | 1.53 | 0.22 |  | 0.798 |
| ≤60 | 90 | 36(40.0) | 54(60.0) |  |  | 4.41±2.54 |  |
| >60 | 98 | 48(49.0) | 50(51.0) |  |  | 4.51±2.74 |  |
| Gender |  |  |  | 0.003 | 0.96 |  | 0.426 |
| Male | 119 | 53(44.5) | 66(55.3) |  |  | 4.58±2.64 |  |
| Female | 69 | 31(44.9) | 38(55.1) |  |  | 4.36±2.66 |  |
| Tumor location |  |  |  | 1.83 | 0.18 |  | 0.082 |
| Head-neck | 113 | 55(48.7) | 58(51.3) |  |  | 4.50±2.74 |  |
| Body-tail | 75 | 29(38.7) | 46(61.3) |  |  | 4.41±2.51 |  |
| Lymph node metastasis |  |  |  | 21.75 | **<0.001^*^** |  | **<0.001^*^** |
| Yes |  | 57(62.0) | 35(38.0) |  |  | 5.40±2.60 |  |
| No |  | 27(28.1) | 69(71.9) |  |  | 3.56±2.36 |  |
| Distant metastasis |  |  |  |  | 0.087 |  | 0.058 |
| Yes | 3 | 3(100.0) | 0(0.0) |  |  | 7.33±1.16 |  |
| No | 185 | 81(43.8) | 104(56.2) |  |  | 4.42±2.63 |  |
| TNM stage |  |  |  | 15.24 | **0.002^*^** |  | **<0.001^*^** |
| I | 48 | 15(31.3) | 33(68.8) |  |  | 3.81±2.17 |  |
| II | 119 | 52(43.7) | 67(56.3) |  |  | 4.29±2.75 |  |
| III | 18 | 14(77.8) | 4(22.2) |  |  | 6.83±1.51 |  |
| IV | 3 | 3(100.0) | 0(0.0) |  |  | 7.33±1.16 |  |
| Tumor differentiation |  |  |  | 0.10 | 0.748 |  |  |
| I-II | 123 | 56(45.5) | 67(54.5) |  |  |  |  |
| III-IV | 65 | 28(43.1) | 37(56.9) |  |  |  |  |
| Neurovascular invasion |  |  |  | 0.01 | 0.908 |  | 0.455 |
| Yes | 73 | 33(45.2) | 40(54.8) |  |  | 4.35±2.83 |  |
| No | 115 | 51(44.3) | 64(55.7) |  |  | 4.64±2.32 |  |

| **Table S2.** Univariate and multivariate Cox proportional hazard analyses of patients with PDAC (Retrospective cohort) | | | | | | | |
| --- | --- | --- | --- | --- | --- | --- | --- |
|  | Univariate analysis | | |  | Multivariate analysis | | |
| Variable | HR | 95%CI | *P-*value |  | HR | 95%CI | *P*-value |
| Age | 1.272 | 0.910-1.778 | 0.159 |  |  |  |  |
| Gender | 0.851 | 0.599-1.209 | 0.367 |  |  |  |  |
| Tumor location | 1.148 | 0.819-1.608 | 0.423 |  |  |  |  |
| T stage |  |  | 0.382 |  |  |  |  |
| T1 | Reference |  |  |  |  |  |  |
| T2 | 1.359 | 0.498-3.712 | 0.549 |  |  |  |  |
| T3 | 1.076 | 0.389-2.977 | 0.887 |  |  |  |  |
| N stage |  |  | **<0.001^*^** |  |  |  | **<0.001^*^** |
| N0 | Reference |  |  |  | Reference |  |  |
| N1 | 1.906 | 1.331-2.730 | **<0.001^*^** |  | 2.007 | 1.372-2.935 | **<0.001^*^** |
| N2 | 2.261 | 1.292-3.958 | **0.004^*^** |  | 2.618 | 1.396-4.911 | **0.003^*^** |
| M stage | 2.267 | 0.719-7.142 | 0.162 |  |  |  |  |
| TNM stage |  |  | 0.067 |  |  |  |  |
| I | Reference |  |  |  |  |  |  |
| II | 1.294 | 0.859-1.949 | 0.218 |  |  |  |  |
| III | 2.038 | 1.111-3.740 | **0.021^*^** |  |  |  |  |
| IV | 2.881 | 0.877-9.465 | 0.081 |  |  |  |  |
| Neurovascular invasion | 1.228 | 0.875-1.722 | 0.235 |  |  |  |  |
| Tumor differentiation | 2.009 | 1.423-2.836 | **<0.001^*^** |  | 2.638 | 1.396-4.911 | **<0.001^*^** |
| TIPE3 expression | 1.919 | 1.371-2.685 | **<0.001^*^** |  | 1.585 | 1.103-2.278 | **0.013^*^** |
| HR, hazard ratio; CI, confidence interval | | | | | | | |

| **Table S3.** Correlation between TIPE3 expression and clinical characteristics of patients with PDAC (Prospective cohort) | | | | | | | |
| --- | --- | --- | --- | --- | --- | --- | --- |
| Characteristic | Number | TIPE3 expression | | χ2 value | *P*-value | TIPE3 Staining Score | *P*-value |
|  |  | High (%) | Low (%) |  |  |  |  |
| Age (years) |  |  |  | 0.001 | 1.000 |  | 0.470 |
| ≤60 | 27 | 11(40.7) | 16(59.3) |  |  | 4.11±2.41 |  |
| >60 | 39 | 16(41) | 23(59) |  |  | 4.56±2.54 |  |
| Gender |  |  |  | 0.884 | 0.451 |  | 0.553 |
| Male | 37 | 17(47.2) | 20(54.1) |  |  | 4.54±2.66 |  |
| Female | 29 | 10(33.3) | 19(65.5) |  |  | 4.17±2.25 |  |
| Tumor location |  |  |  | 1.306 | 0.318 |  | 0.305 |
| Head-neck | 36 | 17(47.2) | 19(52.8) |  |  | 4.67±2.60 |  |
| Body-tail | 30 | 10(33.3) | 20(66.7) |  |  | 4.03±2.33 |  |
| Lymph node metastasis |  |  |  | 3.512 | 0.080 |  | **0.030^*^** |
| Yes | 30 | 16(53.3) | 14(46.7) |  |  | 5.01±2.11 |  |
| No | 36 | 11(30.6) | 25(69.4) |  |  | 3.78±2.63 |  |
| Distant metastasis |  |  |  | 0.020 | 0.639 |  | 0.469 |
| Yes | 4 | 1(25.0) | 3(75.0) |  |  | 4.44±2.52 |  |
| No | 62 | 26(41.9) | 36(58.1) |  |  | 3.50±1.73 |  |
| TNM stage |  |  |  | 6.256 | 0.100 |  | 0.062 |
| I | 20 | 5(25.0) | 15(75.0) |  |  | 3.35±2.85 |  |
| II | 37 | 17(45.9) | 20(54.1) |  |  | 4.81±2.26 |  |
| III | 5 | 4(80.0) | 1(20.0) |  |  | 6.00±1.41 |  |
| IV | 4 | 1(25.0) | 3(75.0) |  |  | 3.50±1.73 |  |
| Tumor differentiation |  |  |  | 2.742 | 0.122 |  | 0.101 |
| I-II | 42 | 14(33.3) | 28(66.7) |  |  | 4.00±2.43 |  |
| III-IV | 24 | 13(54.2) | 11(45.8) |  |  | 5.04±2.48 |  |
| Neurovascular invasion |  |  |  | 0.299 | 0.624 |  | 0.110 |
| Yes | 32 | 12(37.5) | 20(62.5) |  |  | 3.88±2.81 |  |
| No | 34 | 15(44.1) | 19(55.9) |  |  | 4.85±2.05 |  |

| **Table S4.** Univariate and multivariate Cox proportional hazard analyses of patients with PDAC (Prospective cohort) | | | | | | | |
| --- | --- | --- | --- | --- | --- | --- | --- |
|  | Univariate analysis | | |  | Multivariate analysis | | |
| Variable | HR | 95%CI | *P-*value |  | HR | 95%CI | *P*-value |
| Age | 1.016 | 0.987-0.987 | 0.280 |  |  |  |  |
| Gender | 0.910 | 0.511-1.621 | 0.749 |  |  |  |  |
| Tumor location | 0.783 | 0.437-1.401 | 0.410 |  |  |  |  |
| T stage |  |  | 0.837 |  |  |  |  |
| T1 | Reference |  |  |  |  |  |  |
| T2 | 1.668 | 0.221-12.301 | 0.616 |  |  |  |  |
| T3 | 1.488 | 0.198-11.171 | 0.699 |  |  |  |  |
| N stage |  |  | **0.027^*^** |  |  |  |  |
| N0 | Reference |  |  |  |  |  |  |
| N1 | 2.017 | 1.091-3.730 | **0.025^*^** |  |  |  |  |
| N2 | 3.061 | 1.112-8.426 | **0.030^*^** |  |  |  |  |
| M stage | 3.292 | 1.140-9.506 | **0.028^*^** |  |  |  |  |
| TNM stage |  |  | **0.049^*^** |  |  |  |  |
| I | Reference |  |  |  |  |  |  |
| II | 1.130 | 0.859-1.949 | 0.733 |  |  |  |  |
| III | 2.647 | 1.111-3.740 | 0.077 |  |  |  |  |
| IV | 3.979 | 1.224-12.940 | **0.022^*^** |  |  |  |  |
| Neurovascular invasion | 0.645 | 0.355-1.172 | 0.645 |  |  |  |  |
| Tumor differentiation | 3.169 | 1.748-5.743 | **<0.001^*^** |  | 2.666 | 1.444-4.922 | **0.002^*^** |
| TIPE3 expression | 2.576 | 1.432-4.632 | **0.002^*^** |  | 2.078 | 1.132-3.815 | **0.018^*^** |
| HR, hazard ratio; CI, confidence interval | | | | | | | |

**Table S5.** Baseline characteristics of patients with PDAC (Retrospective cohort)

| Characteristic | Number (%)/Mean±SD |
| --- | --- |
| Age (years) |  |
| Median | 62.0±10.9 |
| Range | 34~85 |
| Gender |  |
| Male | 119(63.3) |
| Female | 69(36.7) |
| Risk factor analysis |  |
| Smoke |  |
| Yes | 23(12.3) |
| No | 165(87.8) |
| Drink |  |
| Yes | 20(10.6) |
| No | 168(89.4) |
| Diabetes |  |
| Yes | 17(9.0) |
| No | 171(91.0) |
| Tumor location |  |
| Head-neck | 113(60.1) |
| Body-tail | 75(39.9) |
| T stage |  |
| T1 | 6(3.2) |
| T2 | 103(54.8) |
| T3 | 79(42.0) |
| N stage |  |
| N0 | 96(51.1) |
| N1 | 74(39.4) |
| N2 | 18(9.6) |
| M stage |  |
| M0 | 185(98.4) |
| M1 | 3(1.6) |
| AJCC TNM stage |  |
| I | 48(25.5) |
| II | 119(63.3) |
| III | 18(9.6) |
| IV | 3(1.6) |
| Tumor differentiation |  |
| I-II | 123(65.4) |
| III-IV | 65(34.6) |
| Neurovascular invasion |  |
| Yes | 73(38.8) |
| No | 115(61.2) |
| TIPE3 expression in tumor |  |
| High | 84(44.7) |
| Low | 104(55.3) |
| TIPE3 expression in normal |  |
| High | 8(5.8) |
| Low | 131(94.2) |

**Table S6.** Baseline characteristics of patients with PDAC (Prospective cohort)

| Characteristic | Number (%)/Mean±SD |
| --- | --- |
| Age (years) |  |
| Median | 64.1±10.8 |
| Range | 38~90 |
| Gender |  |
| Male | 37 (56.1) |
| Female | 29 (43.9) |
| Risk factor analysis |  |
| Smoke |  |
| Yes | 7(10.6) |
| No | 59(89.4) |
| Drink |  |
| Yes | 9(13.6) |
| No | 57(86.3) |
| Diabetes |  |
| Yes | 7(10.6) |
| No | 59(89.4) |
| Tumor location |  |
| Head-neck | 36(54.5) |
| Body-tail | 30(45.5) |
| T stage |  |
| T1 | 2(3.0) |
| T2 | 37(56.1) |
| T3 | 27(40.9) |
| N stage |  |
| N0 | 36(54.5) |
| N1 | 25(37.9) |
| N2 | 5(7.6) |
| M stage |  |
| M0 | 62(93.9) |
| M1 | 4(6.1) |
| AJCC TNM stage |  |
| I | 20(30.3) |
| II | 37(56.1) |
| III | 5(7.6) |
| IV | 4(6.1) |
| Tumor differentiation |  |
| I-II | 42(63.6) |
| III-IV | 24(36.4) |
| Neurovascular invasion |  |
| Yes | 32(48.5) |
| No | 34(51.5) |
| TIPE3 expression in tumor |  |
| High | 27(40.9) |
| Low | 39(59.1) |
| TIPE3 expression in normal |  |
| High | 7(10.6) |
| Low | 42(63.6) |

| **Table S7.** The primers used for qPCR analysis | |
| --- | --- |
| Primer Name | Primer Sequence 5’-3’ |
| TIPE3-F | CAGCATGGATTCGGATTC |
| TIPE3-R | CGCAAGACTCTTTGAACTA |
| RAC1-F | ATGTCCGTGCAAAGTGGTATC |
| RAC1-R | CTCGGATCGCTTCGTCAAACA |
| GAPDH-F | AACGGATTTGGTCGTATTGGG |
| GAPDH-R | CCTGGAAGATGGTGATGGGAT |
